# Integrating ChromaLIVE™ dye with an AI-powered image analysis for real-time monitoring of human mesenchymal stem cells differentiation

**DOI:** 10.1016/j.bbrep.2025.102174

**Published:** 2025-07-23

**Authors:** Cristina Lopez Serrano, Ibrahim Bilem, Teresa Findley, Ilya Goldberg, Gaétan Laroche, Marie-Christine Durrieu

**Affiliations:** a Univ. Bordeaux, CNRS, Bordeaux INP, CBMN, UMR 5248, Pessac, France; b Laboratoire d’Ingénierie de Surface, Centre de Recherche sur les Matériaux Avancés, Département de Génie des Mines, de la Métallurgie et des Matériaux, Université Laval, Québec, QC, G1V 0A6, Canada; c Axe médecine régénératrice, Centre de Recherche du Centre Hospitalier Universitaire de Québec, Hôpital St-François d’Assise, Québec, QC, G1L 3L5, Canada; d Saguaro Biosciences, La Centrale – Espace Entrepreneurial, Université Laval, Quebec, QC, G1V 0A6, Canada; e ViQI, 315 Meigs Road, Suite A261, Santa Barbara, CA, 93109, USA

**Keywords:** hMSC, Cell differentiation, Real-time cell imaging, Artificial intelligence

## Abstract

Techniques for following the differentiation of human mesenchymal stem cells (hMSCs) in laboratory settings prior to their transplantation into living organisms are essential for progress in tissue engineering and regenerative medicine. In this study, we have used a non-toxic fluorescent dye (ChromaLIVE™) coupled with an artificial intelligence (AutoHCS™) powered image analysis system for real-time monitoring of the differentiation of hMSCs. To validate the performance of this novel Live-Cell Imaging assay, its accuracy was benchmarked to a well-established immunocytochemistry method for studying MSC differentiation into osteoblasts. This innovative method utilizes the distinctive phenotypic signature detected by the non-toxic dye to identify and measure differentiation phenotypes, which were found to align with the expression of osteogenic markers. As a highly sensitive, affordable, non-destructive and scalable kinetic assay, this new technology offers promise as a dependable tool for monitoring stem cell differentiation. By delivering real-time insights into the quality of cell batches, it facilitates prompt adjustments and optimization of culture conditions.

## HIGHLIGHTS

• ChromaLIVE™ is a non-toxic fluorescent dye for cell staining.
• AI-powered imaging with ChromaLIVE™ enable to track hMSC differentiation.
• This technology enables real-time cell tracking while preserving cell viability.

## 1 Introduction

Understanding the differentiation of mesenchymal stem cells (MSCs) into osteoblasts is critical for advancing bone tissue engineering and regenerative medicine. As a highly sensitive, affordable, non-destructive, and scalable kinetic assay, the new technology presented therein holds promise as a reliable tool for monitoring stem cell differentiation, alongside conventional techniques such as qPCR, colorimetric assays (Alizarin Red staining, von Kossa staining, and Alkaline Phosphatase assay), and Western blot analysis [1,2]; [3,4]. While highly specific and sensitive, these assays lack temporal information as they provide end-point data by aquiring a snapshot of the cell state at a predesignated time point. This limitation makes tracking the differentiation process over time both labour and resource intensive.

Stem cells have a unique ability to self-renew and differentiate into various cell types that form adult tissues or organs. The behavior of stem cells, including their self-renewal and differentiation, is influenced by a range of physicochemical factors, such as cell-cell interactions [5], cell-extracellular matrix interactions [6], the topography and stiffness of the matrix [[7], [8], [9]], and cellular signaling triggered by soluble factors like cytokines and growth factors [10]. These complex signals in lineage commitment can lead to differentiation heterogeneity within stem cell populations. Despite advancements in controlling stem cell developmental processes, challenges such as differentiation into unintended lineages, the persistence of undifferentiated stem cells, and variations in differentiation kinetics between cells remain significant barriers to their clinical application in regenerative medicine [11]. In stem cell transplantation therapies, unexpected cell types may arise during differentiation, potentially leading to issues such as tumorigenesis [12]. To meet clinical demands, it is crucial to comprehensively monitor the differentiation of transplanted stem cells into the target lineage, ensuring biosafety and improving therapeutic effectiveness.

Currently, various methods for analyzing lineage-specific marker expression, such as immunocytochemistry, Western blot, flow cytometry, and quantitative polymerase chain reaction (qPCR), are commonly used to track stem cell differentiation [3,4]. However, these traditional methods have limitations due to the destructive steps they require, which compromises the spatial and temporal information within the differentiating cell population, making it difficult to track the lineage of cells from parent to progeny. An alternative to this is bright-field imaging, a non-destructive technique for real-time tracking of biological processes over time. Nevertheless, its low contrast as hindered its broader applicability in the field of stem cell research, as it lacks specificity and sensitivity required to resolve subcellular structures or subtle phenotypic change [[13], [14], [15]]. This limitation becomes particularly challenging when assessing differentiating cells with constantly changing morphology, making precise single-cell-level analysis during in vitro differentiation difficult. These challenges have driven the development of new analytical approaches aimed at enabling highly sensitive, non-destructive, single-cell-level monitoring of stem cell differentiation.

In this study, we utilized a multichromatic non-toxic fluorescent dye, ChromaLIVE™, coupled with an artificial intelligence (AI) powered image analysis system, AutoHCS™, to monitor hMSCs differentiation into osteoblasts through live imaging, offering a dynamic, real-time assessment of cellular events. This mix-and-read dye greatly simplifies the experimental workflow as it does not require any washing step, allowing for continuous tracking of live cells, within a single well, while providing a unique staining pattern that changes consistently along with modifications in cell state [16]. Herein, we aim to explore the potential of ChromaLIVE™ to capture temporal phenotypic changes and investigate how they correlate with cell states at various stages of stem cell differentiation. To establish a reliable reference, specific antibodies markers, commonly applied to assess MSCs differentiation into osteoblasts were used as a ground truth [[17], [18], [19]].

Overall, this innovative approach offers a significant advantage in studying MSC differentiation by providing continuous, real-time data while preserving cell viability. The ability to track and quantify transient phenotypes across the entire differentiation process opens new avenues for studying stem cell biology and optimizing cell culture protocols, ultimately advancing tissue engineering and regenerative medicine therapies.

## 2 Materials and methods

### 2.1 Materials

The non-toxic ChromaLIVE™ dye was supplied by Saguaro Biosciences (Canada). Paraformaldehyde (PFA), Triton X-100, Tween 20 and bovine serum albumin (BSA) were obtained from Sigma-Aldrich (France). Phosphate buffered saline (10 × ) (PBS), trypsin/EDTA (ethylenediaminetetraacetic acid), fetal bovine serum (FBS), Dulbecco's modified Eagle medium (DMEM), DAPI (4′,6-diamidino-2-phenylindole, dihydrochloride), Rhodamine Phalloidin, and goat anti-mouse lgG (H + L) highly cross-adsorbed secondary antibody Alexa Fluor™ 488 were purchased from Thermo Fisher Scientific. Mouse monoclonal antiosteopontin was obtained from Invitrogen. Mouse monoclonal anti-RUNX2 was obtained from Abnova. Bone marrow-derived hMSCs and MSC osteogenic differentiation medium were obtained from Promocell.

### 2.2 Cell culture and live-cell staining with ChromaLIVE™

Primary bone marrow-derived hMSCs were grown on culture flasks in DMEM medium containing 10 % FBS and subcultured with trypsin/EDTA 1x. Cells were incubated in standard cell culture conditions. At passage 5, cells were seeded at a density of 1000 cells per well in Greiner Bio-One glass-bottom 96-well plates with growth medium (DMEM + 10 % FBS). After 24 h of incubation, the medium was replaced by either growth or osteogenic medium, both containing 0.1 % ChromaLIVE™ dye (1:1000 dilution). Medium was changed every third day, each time containing ChromaLIVE™. All experiments were performed in triplicates.

Cells stained with ChromaLIVE™ were cultured for two weeks and imaged every 24 h, except weekends, using ZEISS LSM800 confocal laser scanning microscope, equipped with Zen Blue software (Zeiss, Germany) and a 20× objective. Table 1 provides the imaging settings used to acquire fluorescent images of ChromaLIVE-stained MSCs. ChromaLIVE™ is a multichromatic dye with three excitation/emission wavelength pairs. Briefly, the first excitation wavelength was set at 488 nm, with fluorescence emission collected around 600 nm (ChromaLIVE-488_Yellow) and 700 nm (ChromaLIVE-488_Red). The second excitation wavelength was set at 561 nm, with fluorescence emission collected around 600 nm (ChromaLIVE-561). For each replicate well, five fields of view were captured, resulting in a total of 15 images per condition per time point. It must be pointed out that ChromaLIVE is not a target-specific marker, which accounts for its non-toxic nature. However, it labels multiple organelles and cellular compartments, thereby providing a multiparametric readout of phenotypes at both the cellular and subcellular levels. This enables the detection and quantification of morphological or morpho-functional changes occurring during the differentiation process, which can be analyzed by the AI algorithm.

**Table 1:** Excitation and emission wavelengths for ChromaLIVE™ imaging.

Channel	Excitation wavelength	Emission wavelength
**ChromaLIVE-488_Yellow**	488 nm	550–630 nm
**ChromaLIVE-488_Red**	488 nm	630–750 nm
**ChromaLIVE-561**	561 nm	575–630 nm

### 2.3 High-content imaging data analysis

**Fig. S1:**
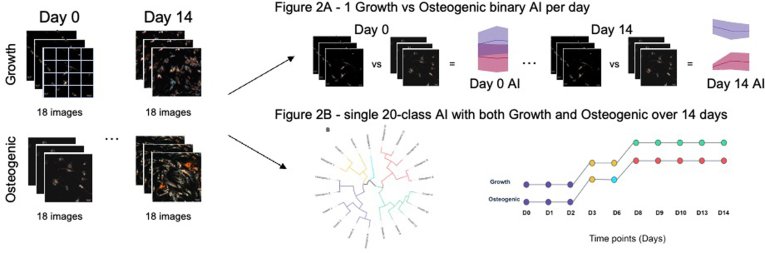
Workflow detailing the number/timepoint of images used for the two training. Images are processed (18 images per condition and timepoint) by tiling into a 4x4 grid resulting in 288 samples per condition and timepoint. Each sample has three channels (which are the three couples of the excitation and emission wavelengths for the dye). Features are extracted using a custom algorithm for each tile and then used to train a series of feature classifiers. For Fig. 2A, a binary AI is trained at each timepoint to distinguish between growth media and osteogenic media resulting in 10 different binary AIs. For Fig. 2B, a single feature classifier is trained on condition + timepoint resulting in predictions on a total of 20 different conditions. These can be plotted in a dendrogram based on euclidean distance between conditions. They can also be redistributed across time to show changes in state.

Time-course images of differentiating MCSs stained with ChromaLIVE™ were analyzed using ViQi's Auto-HCS™ platform. AutoHCS is an automated and unbiased, AI-powered image analysis system that leverages advanced machine learning to enhance the detection of cellular states by agnostically identifying and quantifying subtle cell phenotypes, subcellular structures and molecular markers. This analysis toolkit entirely determines its training parameters using experimental controls rather than user input, which eliminates subjective criteria selection that may bias the assessment of biological responses to applied treatments. At first, binary AI models were trained at each time point on a set of images representing both the control and osteogenic conditions to predict hMSCs commitment towards the osteoblastic lineage. Then, an AI model was trained on all 10 time points for each medium condition to identify phenotypic clusters throughout the 14-day differentiation process. More specifically, there were 18 3-channel images per condition/timepoint for a total of 18 images x 2 conditions x 10 timepoints = 360 images analyzed. Raw tif images (16-bit unsigned pixel planes for each of the 3 ChromaLIVE™ channels) were used in the analysis. These images were tiled into 4x4 tiles, resulting in 360 x 16 = 5760 samples for AI training. Samples were composed of 3 feature vectors (one per channel), with each feature vector containing 2895 numerical image descriptors computed using CharmFeatures (https://gitlab.com/iggman/charm-features/). The sample features were processed by ViQi's proprietary feature classifier auto-trainer (Auto-HCS), which relies on scikit-learn [20] and custom code to perform an algorithm and parameter search to rank/score the features, normalize them, sub-select and weight them (i.e. use soft and hard thresholding) and use them to train feature-based classifiers such as Random Forrest, Support Vector Machines, and WND5 [21], automatically choosing the best performing collection of algorithms and parameters in a 5-fold cross-validation. Finally, cell phenotypes were predicted for each timepoint and plotted as mean and standard deviation. Fig. S1 shows a schematic description of the AI-driven analysis workflow.

### 2.4 Immunocytochemistry analysis

Cells for immunocytochemistry analysis were cultured as described previously, with either growth or osteogenic medium, and fixed after 7 days of incubation. Cells were fixed in a 4 % PFA solution for 15 min at 4 °C, then permeabilized with 0.5 % TritonX-100 for 5 min and saturated for 1 h with 3 % BSA. Afterwards, the samples were incubated overnight at 4 °C with primary antibodies, each applied to a separate well (mouse monoclonal anti-Runx2 at a dilution of 2.5 g mL^−1^ and mouse monoclonal anti-osteopontin at a dilution of 2.5 μg mL^−1^, in 1 % BSA/PBS). Subsequently, each well was incubated with the secondary antibody (goat anti-mouse lgG (H + L) highly cross-adsorbed secondary antibody Alexa Fluor™ 488), diluted at 5 μg mL^−1^ in 1 % BSA/PBS for 1 h at 37 °C. Cell cytoskeleton was labeled with rhodamine phalloidin for 1 h at 37 °C. Finally, cell nuclei were stained using DAPI at 1:1000 dilution. All samples were washed with PBS containing 0.05 % Tween 20 after each incubation steps.

Stained cells were examined using the previously described confocal laser scanning. Images for protein expression quantification were captured at 20× magnification, followed by analysis using Image J open-source software. The expression of the differentiation markers was evaluated by quantifying the fluorescence intensity in the nuclear region of 70–100 cells per condition in the case of RUNX2 and for 200–300 cells in the case of osteopontin (SPP1). Results were expressed as mean ± standard deviation. Statistical significance was evaluated via t-tests with Welch's correction for unequal variance, with GraphPad Prism 8.0.1.

### 2.5 Alizarin Red staining

The extent of mineralization was qualitatively assessed with Alizarin red staining. Briefly, cells were fixed with PFA 4 % and rinsed with PBS. Cells were incubated with 2 % Alizarin red solution for 10 min at room temperature. After incubation, cells were rinsed abundantly with ultrapure water until the rinsing solution became transparent and imaged to assess mineralization.

## 3 Results and discussion

### 3.1 Predicting stem cell differentiation through real-time phenotypic profiling

**Fig. 1:**
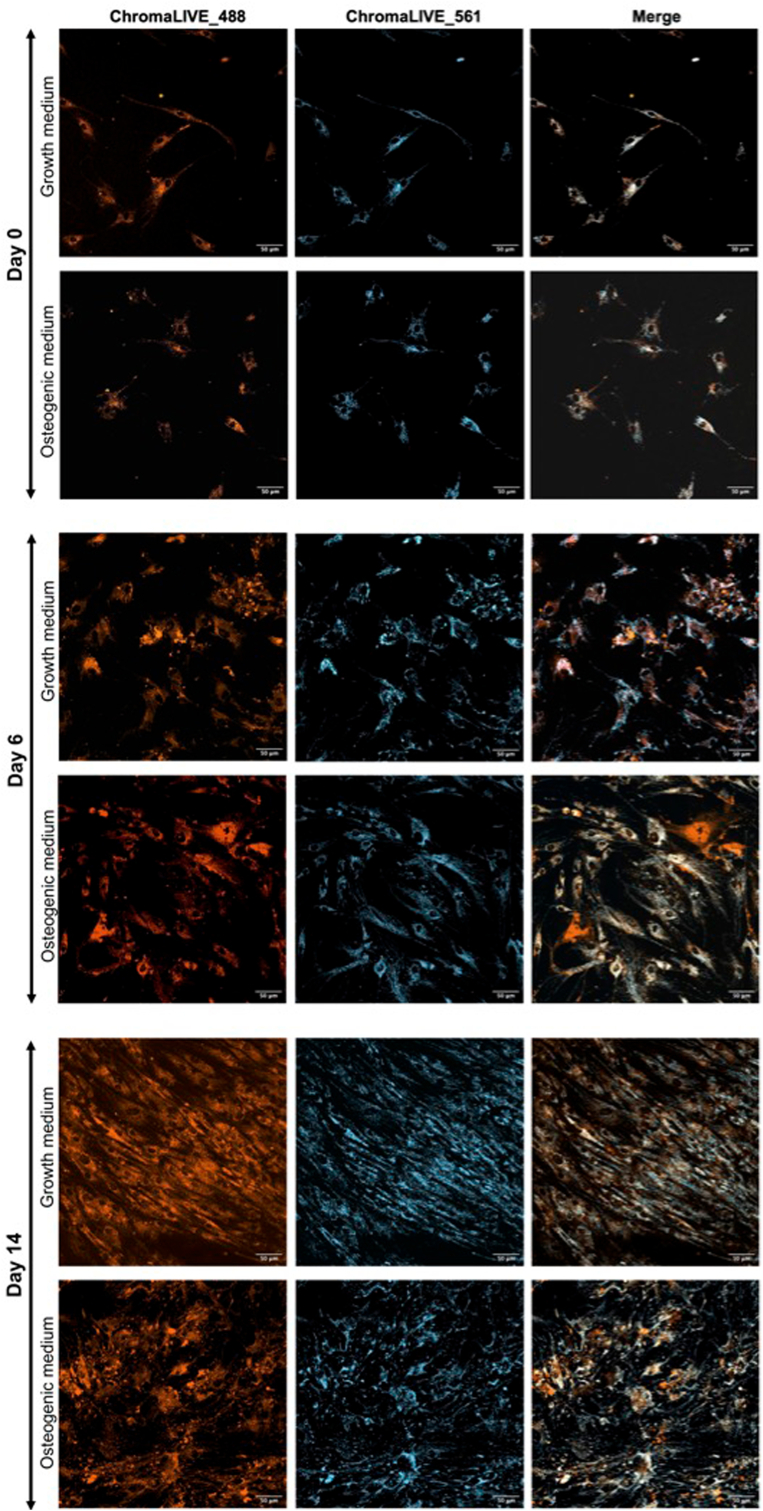
Fluorescence microscopy used to perform the osteogenic differentiation quantitative analysis images of live hMSCs cultured for 14 days in osteogenic or growth medium and stained with ChromaLive™. ChromaLive-488 and ChromaLive-561 are shown in Orange and Cyan, respectively. Images were acquired at Day 0 (day of osteogenic medium addition), Day 6 and Day 14 (scale bar: 50 μm).

**Fig. 2:**
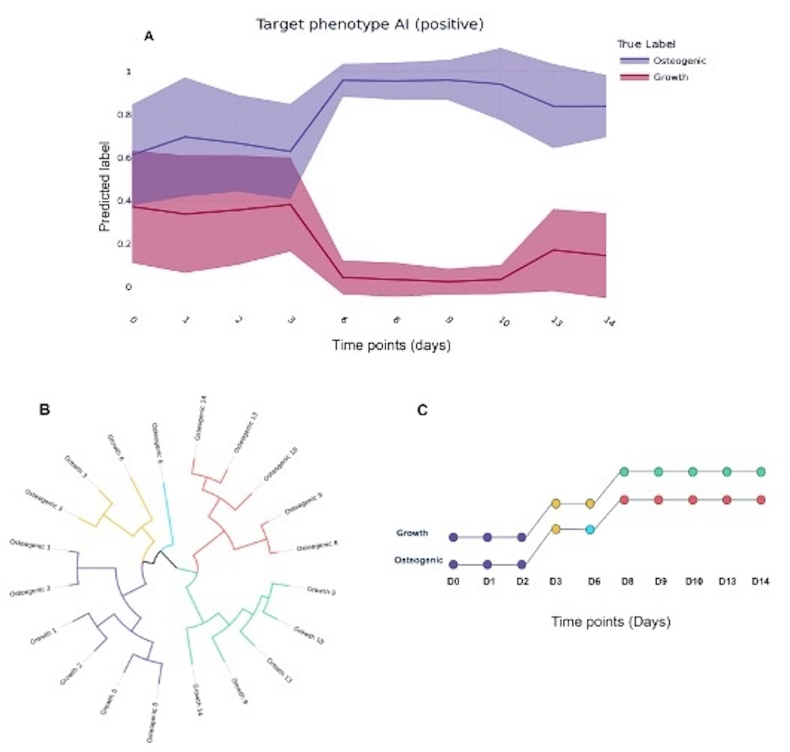
(A) Binary AI trained at each time point on growth medium versus osteogenic, using ViQi's Auto-HCS™ analysis platform. A unique and fully distinguishable phenotype was detected by day 6 in osteogenic medium, predicting the differentiation of hMSCs towards the osteoblastic lineages. (B) and (C) Quantitative clustering analysis of hMSCs differentiation. Circular hierarchical clustering analysis dendrogram (B) and Time-course phenotypic state analysis (C) grouping hMSCs states into 5 distinct phenotypic clusters, dependent on both time and culture conditions. At the early stage, hMSCs under growth and osteogenic conditions cluster together (Purple). At the intermediate stage, transient phenotypes appear (yellow/blue) and become condition-specific by day 6. At the late stage, hMSCs were classified into two fully distinguishable and persistent clusters, reflecting the respective culture conditions (green/red).

**Fig. S2:**
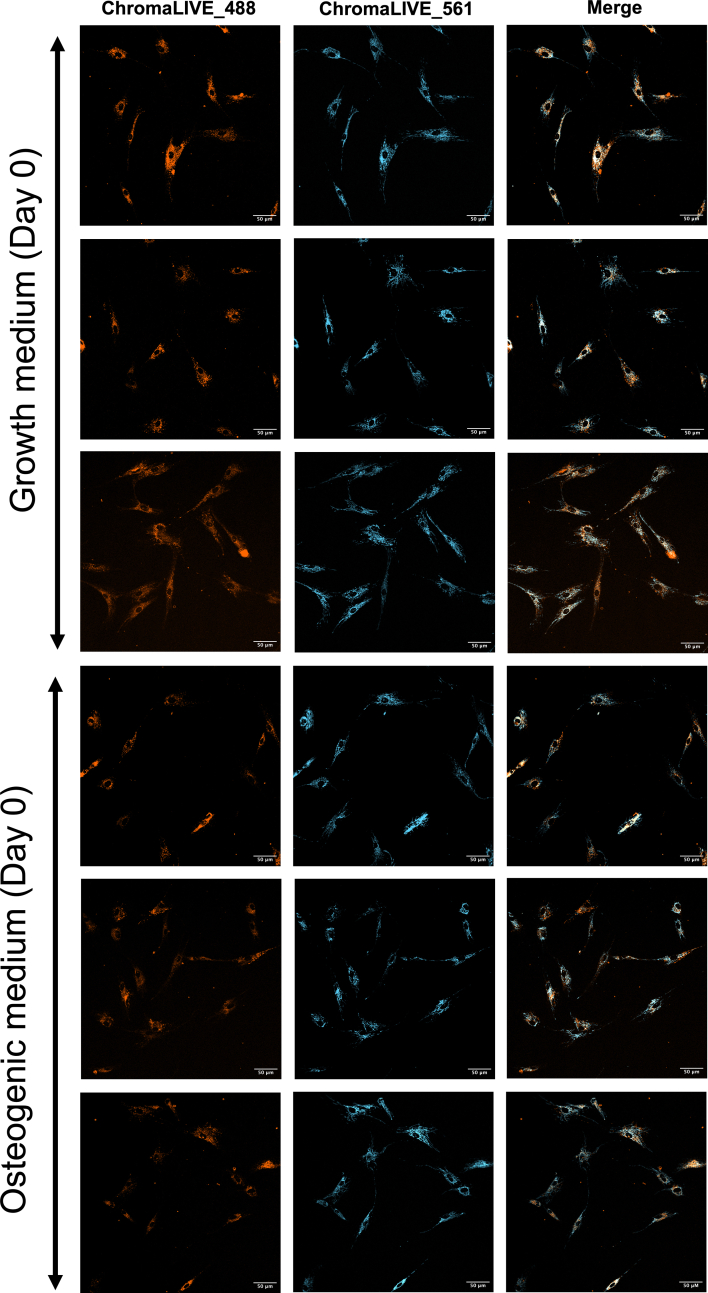
Three typical fluorescence microscopy images acquired at Day 0 (day of osteogenic medium addition) used to perform the osteogenic differentiation quantitative analysis images of live hMSCs in osteogenic or growth medium and stained with ChromaLive™. ChromaLive-488 and ChromaLive-561 are shown in Orange and Cyan, respectively (scale bar: 50 μm).

**Fig. S3:**
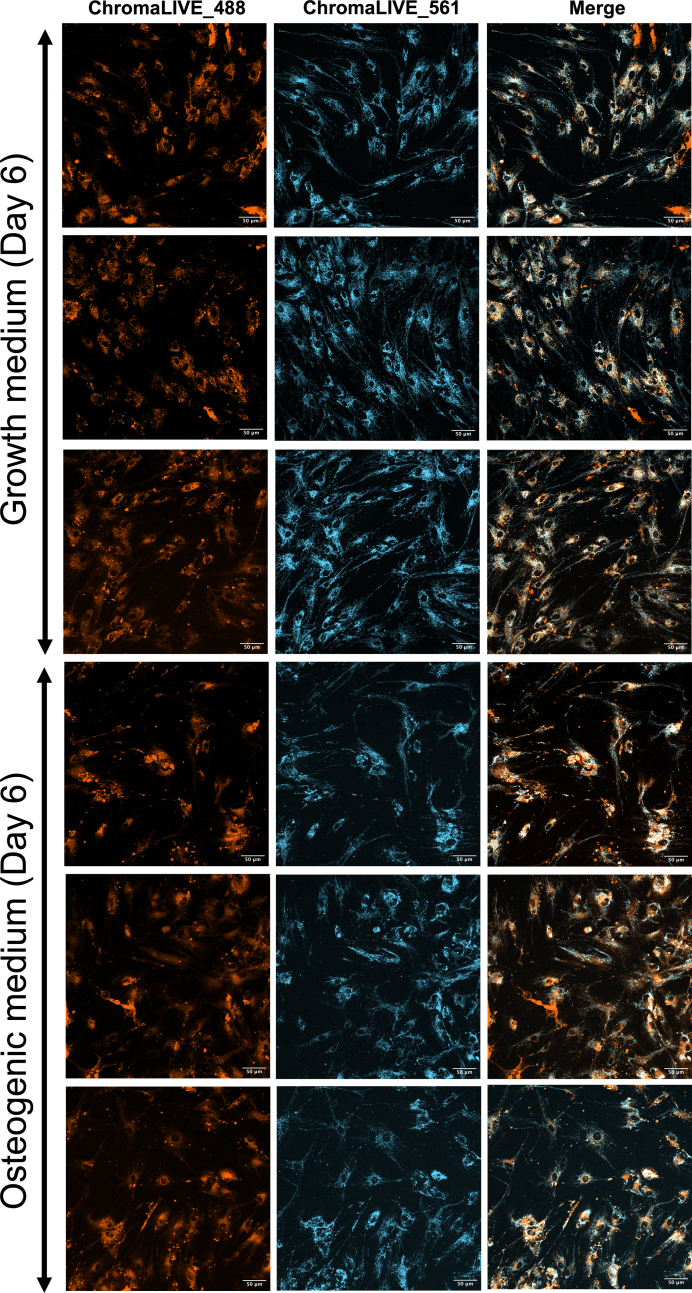
Three typical fluorescence microscopy images acquired after 6 days of culture, used to perform the osteogenic differentiation quantitative analysis images of live hMSCs in osteogenic or growth medium and stained with ChromaLive™. ChromaLive-488 and ChromaLive-561 are shown in Orange and Cyan, respectively (scale bar: 50 μm).

**Fig. S4:**
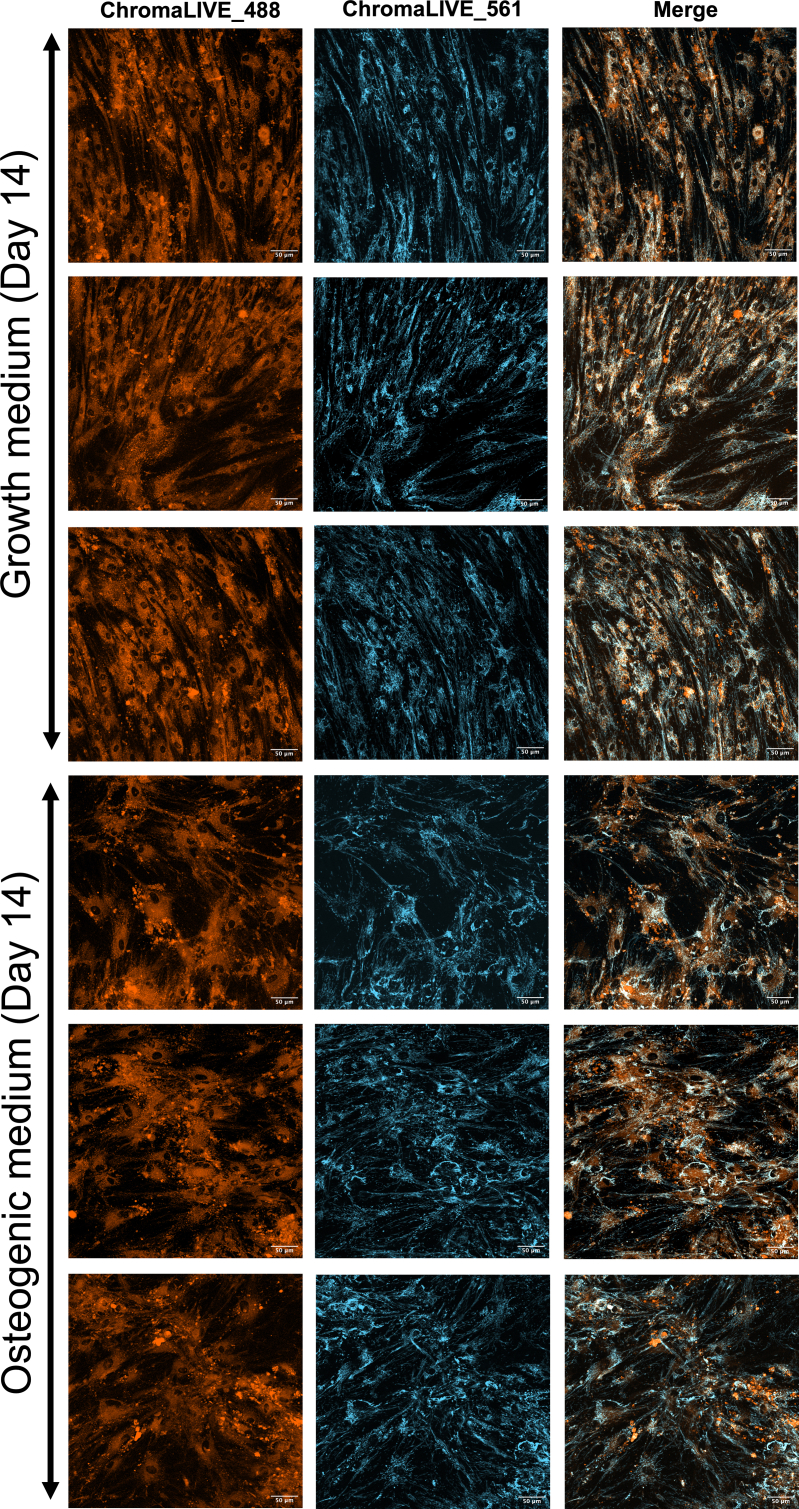
Three typical fluorescence microscopy images acquired after 14 days of culture, used to perform the osteogenic differentiation quantitative analysis images of live hMSCs in osteogenic or growth medium and stained with ChromaLive™. ChromaLive-488 and ChromaLive-561 are shown in Orange and Cyan, respectively (scale bar: 50 μm).

Data analysis of live-cell kinetic measurements using ChromaLIVETM revealed a progressive and time-dependent divergence in phenotypic profiles between hMSCs exposed to growth and osteogenic media. Fig. 1 depicts representative images of ChromaLIVE-stained hMSCs, where notable differences in cell morphology and staining pattern were observed across various stages of the differentiation process (Day 0, Day 6 and Day 14). Due to space limitations, three representative images for each timepoint are presented in Figs. S2, S3, and S4, allowing the reader to better visualize the data used for the quantitative analysis shown in Fig. 2.

Custom feature extraction was computed on acquired images resulting in over 2000 image features represented as vectors and arrays. These features were divided by condition of the well (osteogenic versus growth media) and then used to train a feature classifier. ViQi's custom software runs a cross validation to determine the best feature classification to use and then trains a model to distinguish between conditions. Fig. 2A shows an AI model trained per timepoint and used to predict validation sets of these images. Specifically, it shows the distribution of predictions per sample at each timepoint, separated by true class (osteogenic or growth). There were 18 images per condition/timepoint total. Quantitative analysis demonstrated that the trained AI classifier was unable to distinguish phenotypic states between the two conditions during the early timepoints (Day 0–3) (Fig. 2A). However, phenotypic differences became increasingly pronounced from Day 3, reaching full distinguishability by Day 6, which persisted until Day 14 (Fig. 2A).

Phenotypic clustering analysis, presented as a dendrogram (Fig. 2-B) or time-course phenotypic state (Fig. 2-C), was consistent with these findings, revealing that hMSCs phenotypes progressively transitioned from an early-stage phenotype (0–2 days) to intermediate phenotypes (Day 3–6) and finally to late phenotypes (Day 8–14). At early stages, hMSCs from both conditions belonged to the same phenotypic cluster. However, during the intermediate stages, two distinct transient phenotypes were observed in induced hMSCs, while only one transient phenotype was seen in non-induced hMSCs. At the late stages, a new phenotype, specific to each culture condition, emerged and was maintained until Day 14.

### 3.2 Correlating phenotypic profiles with osteogenic markers expression

To validate whether the phenotypic shift observed at Day 6, distinguishing control from osteogenic conditions, immunocytochemistry analysis was performed by investigating the expression of the early osteogenic marker RUNX2 and the late osteoblastic marker SPP1. The transcription factor RUNX2 is one of the earliest markers of osteogenic commitment and is essential for the maturation of MSCs into pre-osteoblasts [17]. As osteoblasts mature, they express other proteins such as osteopontin (SPP1), which is a glycoprotein that plays a critical role in bone remodeling [22]. The expression of these two markers has been abundantly used to demonstrate osteogenic differentiation of MSCs [19,[23], [24], [25], [26], [27], [28], [29], [30], [31], [32], [33], [34], [35], [36], [37], [38]].

**Fig. 3:**
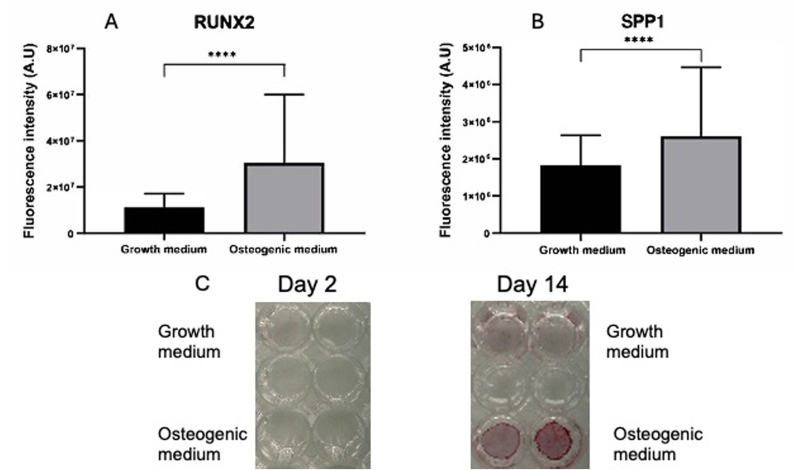
Quantitative analysis of the immunofluorescence of nuclear RUNX2 (A) and SPP1 (B) expression in hMSCS after 1 week of culture in either growth or osteogenic media. (C) Alizarin red staining on cells with growth or osteogenic media after 2 days 14 days of culture.

The results showed that the expression of RUNX2 and SPP1 was significantly higher in hMSCs cultured in osteogenic medium, as compared to those in growth medium, after 1 week (Fig. 3). Osteogenic differentiation at later stages was further confirmed by Alizarin Red staining to evaluate extracellular matrix mineralization, a hallmark of mature osteoblasts. Fig. 3C shows representative images of Alizarin Red staining after 2 and 14 days of culture. While minimal staining was observed at Day 2, extensive calcium deposition was evident by Day 14 in hMSCs cultured in osteogenic medium, confirming their differentiation into a mineralizing osteoblastic phenotype. These findings were further supported by our previous recent studies, in which multiple immunocytochemistry markers and qPCR gene expression analyses consistently indicated the differentiation of hMSCs into bone cells under conditions identical or very similar to those used in the present study ([[26], [27], [28], [29], [30], [31],^39^,^40^]; Padiolleau et al., 2020; [32]).

Altogether, the data presented in Fig. 3 and our previous investigations closely correlates with ChromaLIVETM assay data, revealing the emergence of a distinct and dominant phenotype under osteogenic conditions at Day 6.

In this study, it was demonstrated that ChromaLIVE™ dye, a new generation of non-toxic dyes with unique phenotypic profiling capacity, in combination with advanced AI-powered image analysis, represent a promising novel approach for real-time monitoring of stem cells differentiation. The results demonstrated that stem cell commitment towards the osteoblastic lineage could be predicted as early as Day 6, based on the unique phenotypic fingerprint provided by ChromaLIVE™ (Fig. 2-A). This phenotypic state was shown to be consistent with the differentiation state, assessed through immunocytochemical validation, thus supporting the effectiveness of ChromaLIVE™ in detecting key stages of the differentiation process. To further assess phenotypic changes across the 14-days differentiation period, cluster analysis was performed leading to the identification of 5 distinct phenotypes, driven by both time and culture medium (Fig. 3-A and 3-B). Interestingly, by Day 8, a shift in cell phenotype occurred in the osteogenic condition, suggesting that hMSCs evolved towards a more mature osteoblast phenotype, as compared to Day 6. This phenotypic transition, at Day 8, was also observed in the control condition but the corresponding phenotypic cluster was distinct from that of the osteogenic condition. This suggest that while non-induced hMSCs undergo phenotypic changes over time, they remain clearly distinguishable from differentiating hMSCs.

A key advantage of ChromaLIVE™, as a non-toxic dye, over traditional techniques that require cell fixation or lysis, is its ability to monitor live cells in their native state over prolonged culture periods. This enables a more biologically relevant assessment of stem cell differentiation by providing both spatial and temporal information, offering valuable insights into subtle, transient phenotypic shifts and cellular dynamics. Due to its high sensitivity to detect subtle phenotypes, a cell classifier can be trained, using machine learning algorithms, on ChromaLIVE™ image-based profiles, representing both desired and undesired cell populations as well as cell population at different differentiation stages. This approach offers a potential solution for detecting heterogeneity within stem cell populations throughout a dynamic tracking at a single-cell level during the differentiation process. In addition, as a non-invasive, live imaging approach, enabling continuous monitoring of cell behavior over time within a single well, ChromaLIVE™ not only ensures a more comprehensive understanding of differentiation dynamics but also eliminates the need of multiple samples preparations, thus enhancing both scalability and results reproducibility.

Of note, ChromaLIVE™ can theoretically be used in flow cytometry. However, its utility is highly context dependent. In the present study, the primary objective was to track the dynamic morphological changes associated with stem cell differentiation. Conventional flow cytometry, which primarily relies on quantifying fluorescence intensity, is not well-suited for this purpose. In fact, ChromaLIVE does not bind to a specific cellular target whose expression is up- or down-regulated during differentiation. Instead, it stains multiple intracellular components in a non-specific manner, resulting in a distributed fluorescence signal that does not provide a clear-cut readout of the differentiation state based solely on intensity. ChromaLIVE™ is rather suitable for flow cytometry applications where the goal is to detect strong phenotypes rather than capture subtle phenotypic changes. For example, when cells are exposed to apoptotic stimuli, an increase in the ChromaLIVE_488 signal, associated with a decrease in ChromaLIVE_561 can be observed, providing a meaningful readout of cell health or death in a flow cytometry context.

In microscopy imaging applications, the strength of ChromaLIVE lies in its compatibility with high-content imaging, where thousands of subcellular features per cell can be extracted, including morphology, granularity, texture, signal intensity and colocalization. These multiplexed morphological and spatial information are critical for detecting subtle phenotypic transitions during stem cell differentiation, which are not easily captured by intensity-based measurements alone.

Of interest, the emergence of a new generation of imaging flow cytometers, such as the Cytek®, Amnis®, or ImageStream® X Mark II, which combines the high throughput performance of conventional flow cytometry with the spatial resolution and functional insights of microscopy. These hybrid platforms indeed provide a promising opportunity to leverage the full potential of ChromaLIVE in flow cytometry, by enabling both intensity- and morphological-based analyses.

To the best of our knowledge, the most common approaches used for real-time monitoring of stem cell differentiation are Phase-Contrast and Bright-field microscopy [13,14] or fluorescence imaging, using fluorescent protein reporters [41]. Although, label-free imaging minimizes potential perturbation of cells, it lacks sensitivity and specificity, making it difficult to detect subcellular structure and distinguish subtle phenotypic differences, crucial for stem cell differentiation studies. Moreover, these imaging modalities are prone to artifacts that may affect results interpretation and data quality [15]. On the other hand, the use of fluorescent reporters provides high specificity as it enables tracking of lineage-specific genes expression in real-time. However, it requires genetic modification, which may disturb cell functions, natural cell behavior, differentiation potential or phenotypic features, especially in primary cells know to be difficult to transfect or transduce [42].

This study highlights the unique advantages of ChromaLIVE™, a non-toxic, high-density information dye, combined with ViQI's AutoHCS™ platform, an AI-driven analysis tool that eliminates the potential bias inherent in manual image analysis. This new approach leverages the unique phenotypic signature provided by ChromaLIVE™ to detect and quantify time-sensitive differentiation phenotypes which, in the current study, were shown to correlate with the expression profile of osteogenic markers. As a highly sensitive, cost-effective and scalable kinetic assay, ChromaLIVE™ has the potential to serve as a reliable biological characterization method for real-time monitoring of stem cell differentiation. By providing real-time feedback on cell batches quality, it enables timely intervention and optimization of culture conditions. Moreover, its compatibility with downstream analyses such as immunochemistry, transcriptomics and proteomics, enables comprehensive validation of linage-specific gene and protein expression as well as physiological functions of the differentiated cells.

ChromaLIVE™ also demonstrated robust photostability under repeated kinetic imaging, with no apparent signs of photobleaching or phototoxicity during 1-h intervals over 72-h period (results not shown). In addition, the individual (non-merged) images revealed distinct staining pattern in the 488 nm and 561 nm channels, confirming the absence of bleed-through.

These findings will be validated in future large-scale studies, by exploring additional differentiation lineages, including stem cell differentiation onto adipocytes and chondrocytes. Such studies will further broaden the applicability of ChromaLIVE™, positioning it as a powerful tool for stem cell research.

## CRediT authorship contribution statement

**Cristina Lopez Serrano:** Writing – original draft, Methodology, Investigation, Formal analysis, Data curation, Conceptualization. **Ibrahim Bilem:** Writing – original draft, Validation, Methodology, Investigation, Formal analysis, Data curation, Conceptualization. **Teresa Findley:** Writing – review & editing. **Ilya Goldberg:** Writing – review & editing. **Gaétan Laroche:** Writing – review & editing, Validation, Supervision, Resources, Project administration, Methodology, Investigation, Funding acquisition, Formal analysis, Data curation, Conceptualization. **Marie-Christine Durrieu:** Writing – review & editing, Validation, Supervision, Resources, Project administration, Methodology, Investigation, Funding acquisition, Formal analysis, Data curation, Conceptualization.

## Declaration of competing interest

The authors declare the following financial interests/personal relationships which may be considered as potential competing interests: Gaetan Laroche reports financial support was provided by 10.13039/501100000038Natural Sciences and Engineering Research Council of Canada. Marie-Christine Durrieu reports financial support was provided by 10.13039/501100001665French National Research Agency. Gaetan Laroche reports administrative support was provided by 10.13039/100015427Quebec Centre for Advanced Materials. Ibrahim Bilem reports a relationship with Saguaro Technologies Inc that includes: employment. Teresa Findley reports a relationship with ViQi, Inc. that includes: employment. Ilya Goldberg reports a relationship with ViQi, Inc. that includes: equity or stocks. If there are other authors, they declare that they have no known competing financial interests or personal relationships that could have appeared to influence the work reported in this paper.

## Data Availability

Data will be made available on request.
